# 2D Mathematical Modelling of Overlimiting Transfer Enhanced by Electroconvection in Flow-Through Electrodialysis Membrane Cells in Galvanodynamic Mode

**DOI:** 10.3390/membranes9030039

**Published:** 2019-03-11

**Authors:** Aminat Uzdenova

**Affiliations:** Department of Computer Science and Computational Mathematics, Federal State Budgetary Educational Institution of Higher Education “Umar Aliev Karachai-Cherkess State University”, 369200 Karachaevsk, Russia; uzd_am@mail.ru

**Keywords:** ion-exchange membrane, galvanodynamic mode, electroconvection, chronopotentiogram, current–voltage characteristic, mathematical modelling

## Abstract

Flow-through electrodialysis membrane cells are widely used in water purification and the processing of agricultural products (milk, wine, etc.). In the research and operating practice of such systems, a significant place is occupied by a galvanodynamic (or galvanostatic) mode. 2D mathematical modelling of ion transfer in the galvanodynamic mode requires solving the problem of setting the average current density equal to a certain value, while the current density distribution in the system is uneven. This article develops a 2D mathematical model of the overlimiting transfer enhanced by electroconvection in a flow-through electrodialysis cell in the galvanodynamic mode. The model is based on the system of Navier–Stokes, Nernst–Planck, Poisson equations and equations for the electric current stream function. To set the electric mode we use a boundary condition, relating the electric field strength and current density. This approach allows us to describe the formation of the extended space charge region and development of electroconvection at overlimiting currents. For the first time, chronopotentiograms and current–voltage characteristics of the membrane systems are calculated for the galvanodynamic mode taking into account the forced flow and development of electroconvection. The behaviors of the calculated chronopotentiograms and current–voltage characteristic coincide qualitatively with experimental data. The effects of the electrolyte concentration, forced flow velocity and channel size on the mass transfer at overlimiting currents are estimated.

## 1. Introduction

Membrane systems form the basis of electrodialysis (ED), nano- and microfluidic devices that are used for water purification, agricultural products processing, chemical analysis and many other areas of human activity [1,2,3,4]. According to modern experimental and theoretical studies, the phenomenon of electroconvection significantly affects the transport processes in membrane systems (increases the rate of mass transfer during ED [5,6,7,8,9], reduces or prevents sedimentation [10,11], etc.). Electroconvection is an interfacial phenomenon produced by the action of an external electric field on the electric space charge formed near an ion-selective interface [12]. At overlimiting currents, an extended space charge region (SCR) [13] forms at the depleted membrane surface causing electroconvection that is called electroosmosis of the second kind [14,15]. This kind of electroconvection effectively mixes the depleted solution layer that allows the reduction of diffusion transport limitations [12].

A basis for the mathematical description of overlimiting transfer enhanced by electroconvection in membrane system is the system of Nernst–Planck, Poisson and Navier–Stokes equations. Most of the mathematical models are built for the potentiodynamic (or potentiostatic) mode, which sets the potential drop (PD) between two equipotential planes parallel to the ion-exchange membranes [16,17,18,19,20,21,22,23,24,25,26,27]. However, in research and operating practice (such as chronopotentiometry, impedancemetry and voltammetry) the galvanodynamic (galvanostatic) mode occupies a significant place [28,29,30,31,32,33]. The galvanodynamic mode means that the average current density across the solution/membrane boundaries is kept equal to a given function of time.

The development of mathematical models for the galvanodynamic mode is carried out in several directions:

- The inverse problem method, which suggests that for a given current density the corresponding PD is determined by multiple solutions of the problem for the potentiostatic mode. This method is computationally expensive.
- Decomposition of the system of Nernst–Planck and Poisson equations based on the assumption of local electroneutrality of the electrolyte solution [34,35,36]. In this approach, the distribution of a current density in the system is obtained using the electric current stream function. However, approaches based on the local electroneutrality assumption do not allow taking explicitly into account the effect of the SCR, which is formed at the solution/membrane boundary.
- There is an approach to the galvanodynamic mode modelling, which allows the violation of the electroneutrality of the solution and the formation of the extended SCR to be taken into account. This approach is based on the numerical solution of the Nernst–Planck, Poisson equations with a special boundary condition for the electric potential. Unlike potentiodynamic models [16,17,18,19,20,21,22,23,24,25,26,27], where the potential difference was set, in [37,38,39] the electric field strength at the outer edge of the diffusion layer was specified as an explicit function of the current density for the one-dimensional (1D) case. A similar approach was used for the two-dimensional (2D) case in [40] to study the chronopotentiograms (ChP) of ion-selective microchannel-nanochannel devices with current density uniformly distributed along the border; in [41] to study ChP of heterogeneous ion exchange membranes without taking into account the forced flow.

This article is devoted to the 2D mathematical modelling of overlimiting transfer enhanced by electroconvection in flow-through ED membrane cells in galvanodynamic mode. The proposed model is based on the system of Navier–Stokes, Nernst–Planck and Poisson equations. The galvanodynamic mode is set using the boundary condition connecting the electric field strength and specified current density. The electric current stream function method [34,36,41] is used to take into account the distribution of the current density along the channel. The ChP and current–voltage characteristic (CVC) of the membrane system with taking into account the forced flow and development of electroconvection will be calculated. The effects of the current density and other system parameters on the ChP and intensity of electroconvection will be assessed.

## 2. Mathematical Model

**Figure 1:**
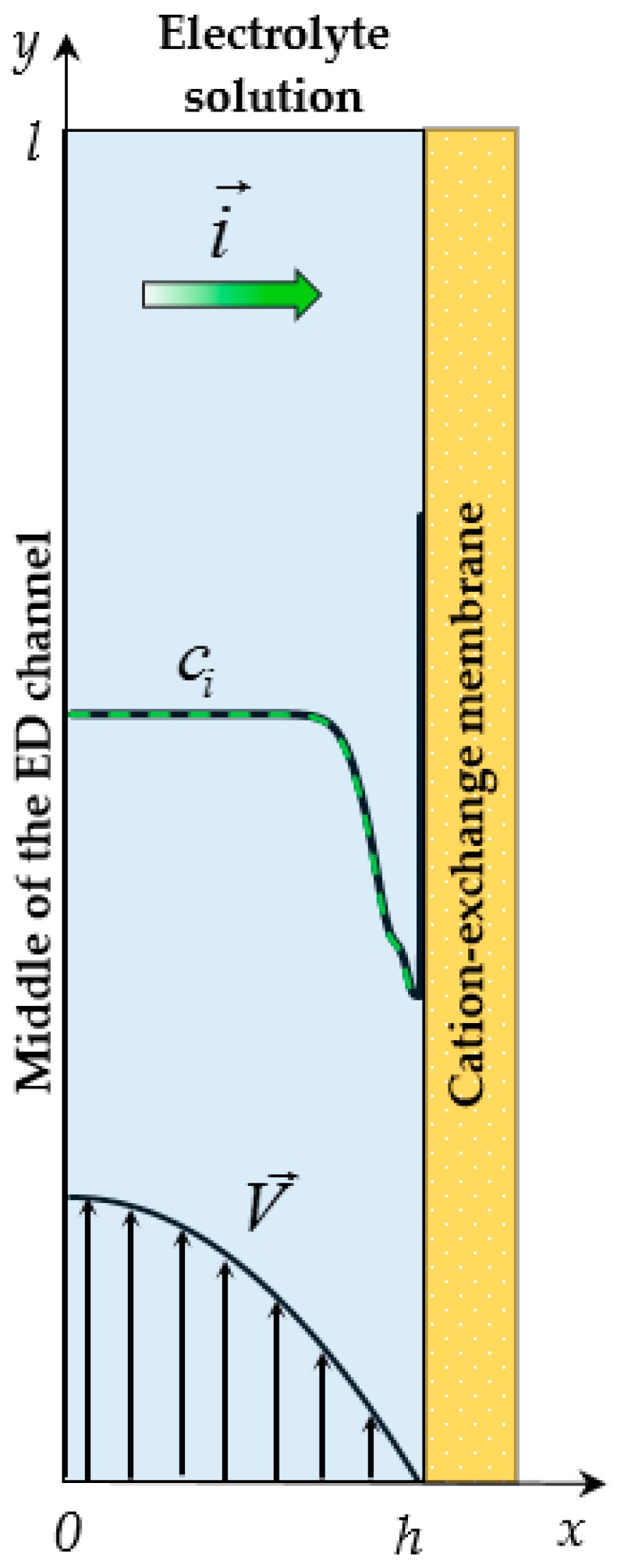
Scheme of the system under consideration: half of the desalination electrodialysis (ED) cell adjacent to the cation-exchange membrane (CEM). Schematic concentration profiles of cations (*c*_1_, solid line) and anions (*c*_2_, dashed line), direction of the electric current $\vec{i}$, forced flow velocity $\vec{V}$ are shown.

The system under consideration is a flow-through ED desalination channel between two ion-exchange membranes. In order to study the overlimiting ChP of an individual membrane and solution layer near its surface, to obtain a fundamental understanding of the mechanisms of processes in this layer, we considered processes in half of the channel at the surface of the cation-exchange membrane (CEM), Figure 1. Reducing the area under consideration to half the channel also significantly decreases the computational complexity of the model. Let *x* and *y* be the transverse and longitudinal coordinates, respectively; *x* = 0 relates to the middle of the ED channel, *x* = *h* is the electrolyte solution/CEM interface; *y* = 0 corresponds to the inlet and *y* = *l* to the outlet of the channel.

### 2.1. Governing Equations

The non-stationary process of transfer of binary electrolyte ions in membrane systems in the absence of chemical reactions, taking into account electroconvection, is written as follows [18,19,20]:

(1) ∂V→∂t+(V→∇)V→=−∇p+1ReΔV→+KelΔφ∇φ, divV→=0,

(2) $${\vec{j}}_{i}=-z_{i}D_{i}c_{i}\nabla \varphi -D_{i}\nabla c_{i}+Pe\;c_{i}\vec{V},\;i=1,\;2,$$

(3) $$\frac{\partial c_{i}}{\partial t}=-\frac{1}{Pe}\;\mathrm{div}\;{\vec{j}}_{i},\;i=1,\;2,$$

(4) $$-\varepsilon \Delta \varphi =z_{1}c_{1}+z_{2}c_{2},$$

(5) $$\vec{i}=z_{1}{\vec{j}}_{1}+z_{2}{\vec{j}}_{2}-\varepsilon \;Pe\;\frac{\partial }{\partial t}(\nabla \varphi ).$$

Equations (1)–(5) are given in dimensionless form. We scale time, *t*, by the value $h/V_{0}$; spatial coordinates, *x* and *y,* by the thickness of the considered region *h* (half of the ED channel thickness); velocity, $\vec{V}$, by the average velocity of the forced flow $V_{0}$; pressure, *p*, by the value $\rho V_{0}^{2}$; concentration of the *i*-th ion, *c_i_*, by the electrolyte concentration in the bulk solution *c*_0_; electric potential, φ, by the value RT/F; individual ion diffusion coefficients, *D*_1_ and *D*_2_, by the electrolyte diffusion coefficient $D=D_{1}D_{2}\left(z_{1}-z_{2}\right)/\left(D_{1}z_{1}-D_{2}z_{2}\right)$; current density, $\vec{i}$, by the value $Dc_{0}F/h$; ion flux ${\vec{j}}_{i}$ by the value $Dc_{0}/h$. Here Re=V0h/ν is the Reynolds number, $Pe=V_{0}h/D$ is the Peclet number, $\varepsilon =RT\varepsilon_{0}\varepsilon_{r}/\left(c_{0}F^{2}h^{2}\right)$ and $K_{el}=\varepsilon_{0}\varepsilon_{r}R^{2}T^{2}/\left(\rho_{0}V_{0}^{2}F^{2}h^{2}\right)$ are the dimensionless parameters; $z_{i}$ is the charge number of the *i*-th ion; *F* is the Faraday constant; R is the gas constant; T is the absolute temperature; $\varepsilon_{0}$ is the dielectric permittivity of vacuum; $\varepsilon_{r}$ is the solution relative permittivity (assumed constant); $\rho_{0}$ is the solution density (assumed constant), ν is the kinematic viscosity.

$\vec{V}$, *p*, ${\vec{j}}_{1}$, ${\vec{j}}_{2}$, *c*_1_, *c*_2_, φ, *i*_x_, *i*_y_ are unknown function of *t*, *x* and *y*. The Navier–Stokes equations, Equations (1), describe the velocity field under the action of the forced flow and the electric body force. The equations of Nernst–Planck, Equations (2), material balance, Equations (3), and Poisson, Equation (4), describe the ion concentration and potential fields. Equation (5) is a formula for the total current density, including the conduction current, ${\vec{i}}_{c}=z_{1}{\vec{j}}_{1}+z_{2}{\vec{j}}_{2}$, and displacement current, ${\vec{i}}_{d}=-\varepsilon \;Pe\;\frac{\partial }{\partial t}(\nabla \varphi )$ [37].

### 2.2. Boundary Conditions

At the channel inlet (*x* ∈ [0, *h*], *y* = 0), the velocity profile is parabolic and satisfies Poiseuille’s law (taking into account the fact that half of the ED channel is considered); the concentration is uniformly distributed along *x*; the condition for the electric potential is obtained from the Equations (2) and (5) considering the zero tangential current density, *i_y_*(*x*, 0, *t*) = 0, (the tangential component of the displacement current, $i_{d\;y}$, is negligible):

(6) $$V_{x}\left(x,0,t\right)=0,\;V_{y}\left(x,0,t\right)=1.5(1-x^{2}),$$

(7) $$c_{i}\left(x,0,t\right)=1,\;i=1,\;2,$$

(8) $$\frac{\partial \varphi }{\partial y}\left(x,0,t\right)=-\frac{1}{z_{1}^{2}D_{1}+z_{2}^{2}D_{2}}\left(z_{1}D_{1}\frac{\partial c_{1}}{\partial y}+z_{2}D_{2}\frac{\partial c_{2}}{\partial y}\right).$$

At the channel outlet (*x* ∈ [0, *h*], *y* = *l*) the velocity profile is again parabolic; the sum of diffusion and migration tangential components of the cation (*i* = 1) and anion (*i* = 2) fluxes is zero; the tangential derivative of the potential is set to be zero:

(9) $$V_{x}\left(x,l,t\right)=0,\;V_{y}\left(x,l,t\right)=1.5(1-x^{2}),$$

(10) $$\left(-\frac{\partial c_{i}}{\partial y}-z_{i}c_{i}\frac{\partial \varphi }{\partial y}\right)\left(x,l,t\right)=0,\;i=1,2,$$

(11) $$\frac{\partial \varphi }{\partial y}\left(x,l,t\right)=0.$$

At *x* = 0, *y* ∈ [0, *l*] (middle of the ED channel) the following conditions are applied:

(12) $$V_{x}\left(0,y,t\right)=0,\;V_{y}\left(0,y,t\right)=1.5,$$

(13) $$c_{i}\left(0,y,t\right)=1,\;i=1,\;2,$$

(14) φ(0,y,t)=0.

At *x* = 1, *y* ∈ [0, *l*] (the solution/membrane interface), the no-slip condition (15) is applied; the counterion concentration, *c*_1_, is set as a constant value *N_c_* greater than the bulk solution concentration, Equation (16) [13]; continuous flow of co-ions, Equation (17); the normal to the membrane surface component of the electric field strength is specified as function of the electric current density, Equation (18) [39]:

(15) $$V_{x}\left(1,y,t\right)=0,\;V_{y}\left(1,y,t\right)=0,$$

(16) $$c_{1}\left(1,y,t\right)=N_{c},$$

(17) $$\left(-D_{2}\frac{\partial c_{2}}{\partial x}-z_{2}D_{2}c_{2}\frac{\partial \varphi }{\partial x}\right)\left(1,y,t\right)=\frac{\left(1-T_{1}\right)}{z_{2}}i_{x}\left(1,y,t\right).$$

(18) $$\frac{\partial \varphi }{\partial x}\left(1,y,t\right)=-\left(\frac{\left(i_{x}+\varepsilon \;Pe\frac{\partial^{2}\varphi }{\partial x\partial t}+z_{1}D_{1}\frac{\partial c_{1}}{\partial x}+z_{2}D_{2}\frac{\partial c_{2}}{\partial x}\right)}{z_{1}^{2}D_{1}c_{1}+z_{2}^{2}D_{2}c_{2}}\right)(1,y,t).$$

Condition (18) was obtained from Equations (2) and (5) [39]. In galvanodynamic mode for 1D case in Equation (18) current density is a function of time, for 2D case the normal current density, *i*_x_, also depends on *y* coordinate. In the formulation of the problem, the average current density, *i*_av_, is used as a parameter determining the electrical mode in the system:

(19) $$i_{av}=\frac{1}{l}\int_{0}^{l}i_{x}(0,y,t)dy=\frac{1}{l}\int_{0}^{l}i_{x}(1,y,t)dy.$$

In the general case the average current density, *i*_av_, is a function of time; in calculating the CVC, it is a linear function of time, *i*_av_ = α*t*, α = *const*; in the calculation of ChP it is a constant, *i*_av_ = *const*; in the case of pulsating currents it is a periodic function of time, etc.

In order to determine the current density distribution along the solution/membrane interface *i_x_*(1,*y*,*t*) (which is required by condition (18)) the electric current stream function method [34,36,41] is used. According to this method, the electric current stream function, η, is determined:

(20) $$i_{x}=\frac{\partial \eta }{\partial y},\;i_{y}=-\frac{\partial \eta }{\partial x}.$$

Then the equation and boundary conditions for η are introduced to the mathematical formulation of the model:

(21) $$\begin{array}{l} \Delta \eta =-\left(\left(z_{1}^{2}D_{1}\frac{\partial c_{1}}{\partial y}+z_{2}^{2}D_{2}\frac{\partial c_{2}}{\partial y}\right)\frac{\partial \varphi }{\partial x}-\left(z_{1}^{2}D_{1}\frac{\partial c_{1}}{\partial x}+z_{2}^{2}D_{2}\frac{\partial c_{2}}{\partial x}\right)\frac{\partial \varphi }{\partial y}\right)+\\ +Pe\left(z_{1}\frac{\partial c_{1}}{\partial y}+z_{2}\frac{\partial c_{2}}{\partial y}\right)V_{x}-Pe\left(z_{1}\frac{\partial c_{1}}{\partial x}+z_{2}\frac{\partial c_{2}}{\partial x}\right)V_{y}+Pe\left(z_{1}c_{1}+z_{2}c_{2}\right)\left(\frac{\partial V_{x}}{\partial y}-\frac{\partial V_{y}}{\partial x}\right), \end{array}$$

(22) $$\frac{\partial \eta }{\partial x}\left(0,y,t\right)=0,\;\frac{\partial \eta }{\partial x}\left(1,y,t\right)=0,\;\eta \left(x,0,t\right)=0,\;\eta \left(x,l,t\right)=i_{av}l.$$

Thus, current density *i*_x_ in boundary condition (18) is determined by formula (20).

The numerical solution of the problem formulated above was obtained by the finite element discretization using the commercially available COMSOL software package.

## 3. Results

### 3.1. Parameters Used in Computations

Most of calculations were performed for ε = 3.05 × 10^−8^, *Pe* = 589, *Re* = 1.07, *K*_el_ = 5.23 × 10^−4^, which correspond to the following system parameters: the thickness of the considered region *h* = 0.5*H*, where *H* = 0.5 × 10^−3^ m is the intermembrane distance; the channel length *l* = 10^−3^ m; the average velocity of forced flow *V_0_* = 3.8 × 10^−3^ m/s; the electrolyte solution density *ρ_0_* = 1002 kg/m^3^; the kinematic viscosity ν = 0.89 × 10^−6^ m^2^/s; the input concentration of the electrolyte solution of NaCl *c*_0_ = 0.1 mol/m^3^; the temperature *T* = 298 K; the diffusion coefficients of cations *D*_1_ = 1.33 × 10^−9^ m^2^/s and anions *D*_2_ = 2.05 × 10^−9^ m^2^/s; the cation transport number in the membrane T_1_ = 0.972 and that in the solution t_1_ = 0.395; the ion charge numbers *z*_1_ = 1, *z*_2_ = −1. To simplify the numerical solution, the ratio of the counterion concentration at the solution/CEM boundary to its value in the bulk solution *N*_c_ was taken as *N*_c_ = 1. This value is less than in real systems [13], however, as Urtenov et al. [42] have shown, when *N*_c_ ≥ 1, the value *N*_c_ does not essentially affect the distribution of concentrations and potential in the extended SCR. In most of the computations, current density *i*_av_/*i*_lim_ = 2, where *i*_lim_ is the dimensionless limiting current density, found by using the Leveque’s equation [12]:

(23) $$i_{\lim}=\frac{1}{T_{1}-t_{1}}\left(1.47{\left(\frac{4h^{2}V_{0}}{lD}\right)}^{1/3}-0.2\right).$$

### 3.2. Chronopotentiogram

**Figure 2:**
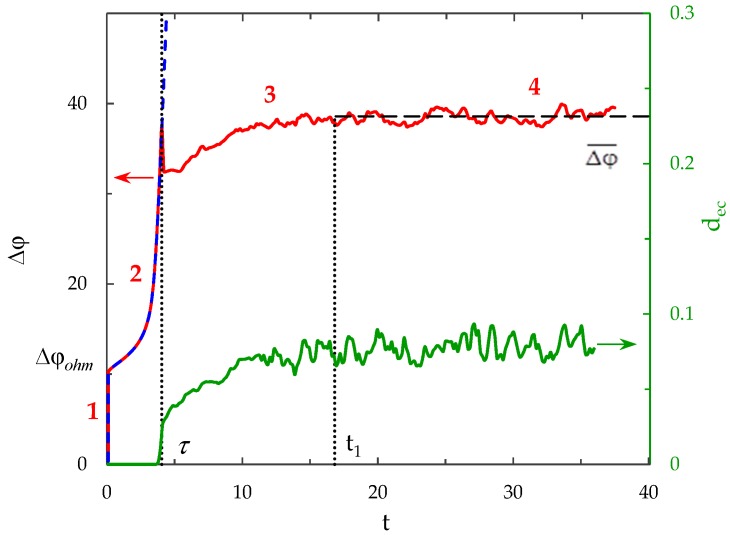
Chronopotentiograms (ChP) calculated with (solid red line) and without (dashed blue line) taking into account electroconvection at *i*_av_/*i*_lim_ = 2. The dotted lines show the transition time *τ* = 3.95 and approximate time of establishing of the quasi-stationary state *t*_1_ ≈ 17. The green line represents the dynamics of the averaged over the channel length thickness of the electroconvective mixing layer, *d*_ec_.

Figure 2 shows the ChP (the dependence of the PD across the system on the time in conditions where a direct current is applied) calculated by the proposed model for *i*_av_/*i*_lim_ = 2. In order to characterize the intensity of electroconvection quantitatively, the averaged over the channel length thickness of the electroconvective mixing layer, *d*_ec_, is also calculated (see Figure 2). The boundary of the electroconvective mixing layer was determined as a point at which the difference in the root-mean-square value of the velocity in calculations with and without taking into account electroconvection exceeds 5% of the average forced flow velocity, *V*_0_, (by analogy with [43], in which for a model without forced flow 20% of the maximum root-mean-square velocity is used as a threshold value).

**Figure 3:**
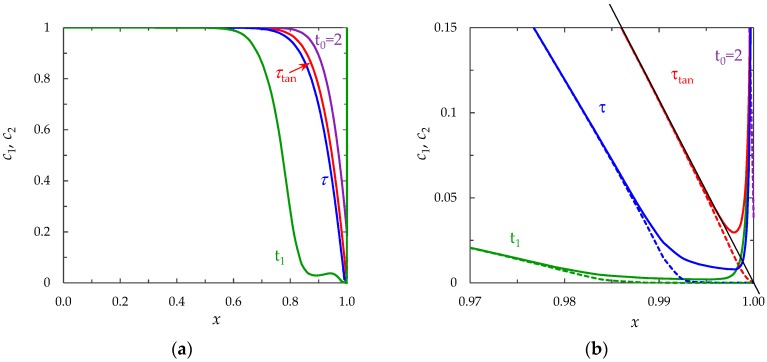
(**a**) Concentration profiles of cations (*c*_1_, solid lines) and anions (*c*_2_, dashed lines) in section *y* = 0.9*l* at different instants of time: *t*_0_ = 2, *τ*_tan_ = 3.15, *τ* = 3.95, *t*_1_ = 17, calculated at *i*_av_/*i*_lim_ = 2; (**b**) enlarged fragment of (**a**).

**Figure 4:**
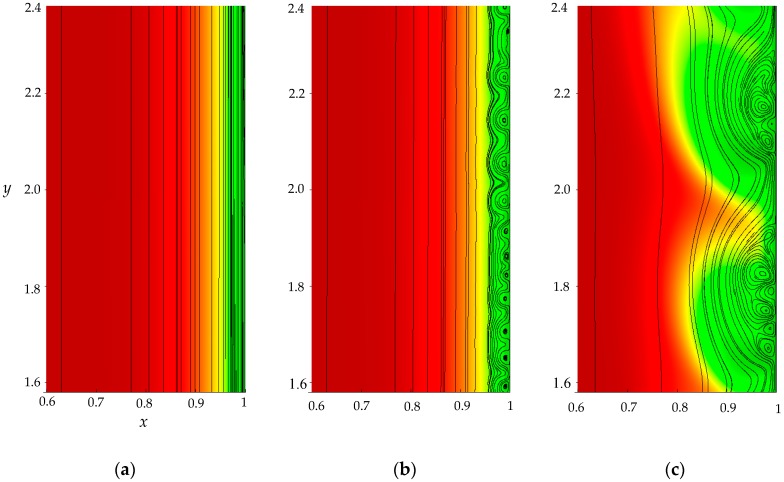
Distribution of cation concentration (the magnitude is shown by different colors), solution streamlines (black lines) in the area at the membrane surface, *i*_av_/*i*_lim_ = 2, *τ*_tan_ = 3.15 (**a**), *τ* = 3.95 (**b**), *t*_1_ = 16.72 (**c**).

The calculated ChP consists of the following sections:

(1) The sharp increase in the PD to the value $\Delta \varphi_{ohm}$ (*t* < 3 × 10^−5^), due to the initial ohmic resistance of the solution. The initial ohmic PD, $\Delta \varphi_{ohm}$, can be estimated by the formula (24) obtained from Equations (2), (5), (23):
  (24) $$\Delta \varphi_{ohm}=\frac{1.47{\left(4h^{2}V_{0}/LD\right)}^{1/3}-0.2}{2(T_{1}-t_{1})(z_{1}^{2}D_{1}+z_{2}^{2}D_{2})}\frac{i_{av}}{i_{\lim}}.$$
(2) The monotonous growth of the PD caused by electrodiffusion processes (3 × 10^−5^ ≤ *t* ≤ *τ*). This section begins with the slow growth of the PD associated with the depletion of the concentration of the electrolyte solution in the region near the membrane surface. Over time, the concentration approaches zero and the growth rate of the PD increases. When the tangent to the electrolyte concentration profile approaches zero at *x* = 1 (*τ*_tan_ = 3.15) the extended SCR is starting to form at the outer edge of the quasi-equilibrium electric double layer (curves τ_tan_, Figure 3). At *t* = τ_tan_ the extended SCR is localized at the relatively small distance from the solution/membrane interface, where viscous forces suppress the development of electroconvection (Figure 4a). Figure 2 also shows the ChP calculated without taking into account the action of electric force ${\vec{f}}_{e}=\Delta \varphi \nabla \varphi$ (dashed line), that is, without taking into account the development of electroconvection. From Figure 2 it can be seen that the difference in ChP calculated with and without electroconvection appears at time *τ* = 3.95 (transition time). At that point in time, the PD and the thickness of the extended SCR (curves *τ*, Figure 3) reach values sufficient to produce electroconvective vortices which under the action of the forced flow slide along the membrane surface (Figure 4b). Electroconvective vortices mix the electrolyte solution, therefore the ion concentrations increase. Hence, when the thickness of the electroconvective mixing layer, *d*_ec_, increases sharply at *τ*, a sharp decrease in the PD is observed.
(3) The transitional stage of electroconvective flow development (*τ* < *t* < *t*_1_). The growth of the thickness of the electroconvective mixing layer, *d*_ec_, slows down, while the PD increases due to electrodiffusion processes. The increase in the PD causes the increase in the thickness *d*_ec_. The increase in *d*_ec_ slows the growth of the PD. At some point in time (which we denote by *t*_1_), a quasi-stationary state is established. At this state the processes of electrodiffusion and electroconvection balance each other.
(4) The quasi-stationary state (*t* > *t*_1_). Both the PD and thickness *d*_ec_ are saturated and fluctuate relative to fixed values $\bar{\Delta \varphi }$ and $\bar{d_{ec}}$, respectively (Figure 2, Figure 3 and Figure 4c). Quantities $\bar{\Delta \varphi }$ and $\bar{d_{ec}}$ are determined as the time average values at *t* > 20.

### 3.3. Current–Voltage Curve

**Figure 5:**
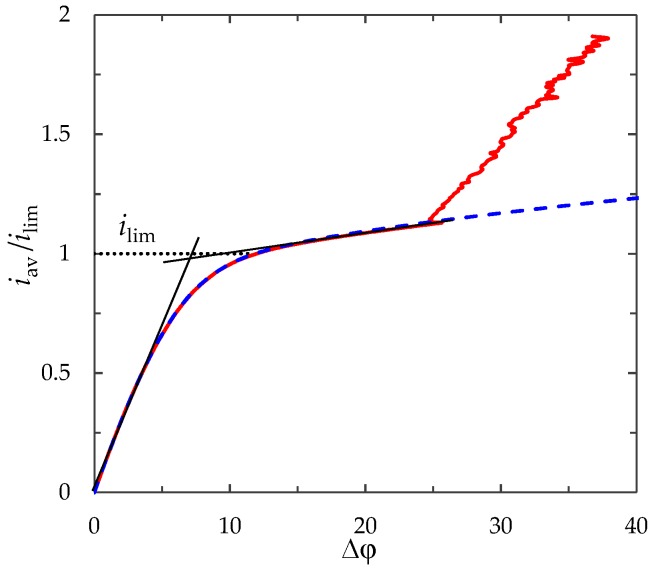
Current–voltage characteristic (CVC) calculated with (solid line) and without (dashed line) taking into account electroconvection. The dotted line shows the limiting current density, *i*_lim_, calculated using Leveque’s Equation (23).

Figure 5 shows the CVC calculated by the galvanodynamic model at $i_{\mathrm{av}}(t)=\alpha t$, α = 0.16. The CVC has a linear initial part. Then, after an intermediary non-linear underlimiting region, the current density grows over the limiting value, *i*_lim_. There is a sloping plateau where the PD smoothly increases with increasing current density. Then the plateau is replaced by a more rapidly increasing region in which oscillations of the PD appear. Then the plateau changes for a steeper region where oscillations of PD occur. Thus, the galvanodynamic model gives qualitatively correct description of experimental CVC curves for the flow-through ED cells [5,7,44]. Note that the limiting current density of the calculated CVC curve, determined by the point of intersection of the tangents drawn to the initial part and to the sloping plateau of the curve is close to *i*_lim_, calculated using Leveque’s Equation (23) (values differ by less than 2%).

As seen from Figure 5, the difference in the CVC calculated with and without taking into account electroconvection appears at *i*_av_ > 1.14*i*_lim_. With an increase in the current density, the difference in the PD at a fixed current density for these two cases is increased. The effect of electroconvection on mass transfer processes depends on the value of the current density. In the next section, we compare the calculation results at a direct current (*i*_av_/*i*_lim_ = 0.9, 1, 1.2,1.5, 2) to eliminate the effect of the current density sweep rate, α.

### 3.4. Effect of the Current Density

**Figure 6:**
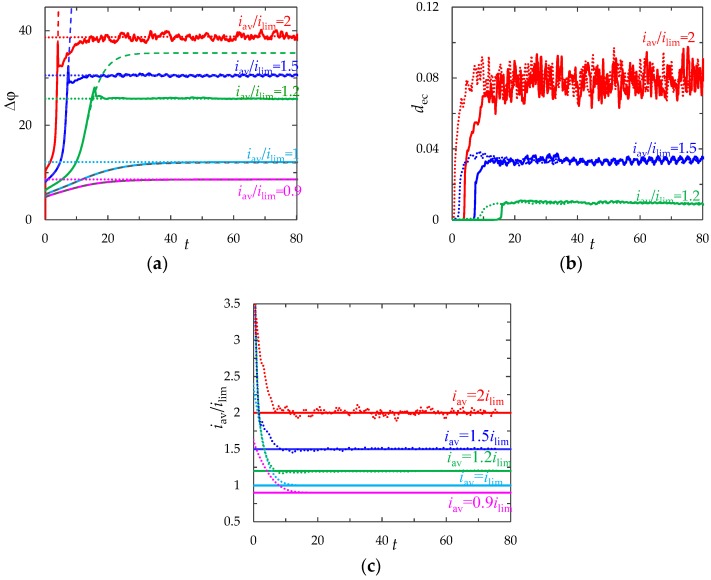
(**a**) ChP calculated with (solid lines) and without (dashed line) taking into account electroconvection; (**b**) the dynamics of the average thickness of the electroconvective mixing layer, *d*_ec_; (**c**) dependence of the average current density on time. The results of calculations in the galvanostatic (solid and dashed lines) and potentiostatic (dotted lines) modes at *i*_av_/*i*_lim_ = 0.9, 1, 1.2, 1.5, 2.

Figure 6a shows ChP calculated with and without taking into account the effect of electric force (${\vec{f}}_{e}=\Delta \varphi \nabla \varphi$) at the following current density values: *i*_av_/*i*_lim_ = 0.9, 1, 1.2,1.5, 2.

The initial ohmic PD of the calculated ChP depends linearly on the current density $\Delta \varphi_{ohm}$ = 5.14*i*_av_/*i*_lim_ (the coefficient before *i*_av_/*i*_lim_ differs from the value of the coefficient in Equation (24) by less than 0.8%).

**Figure 7:**
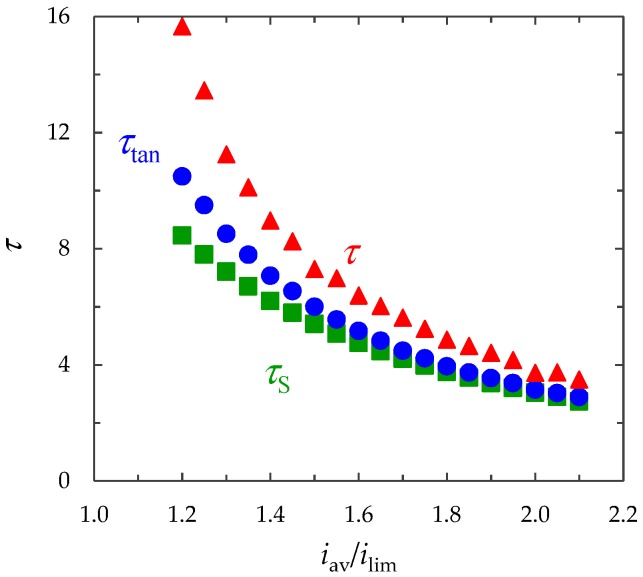
Transient time determined by the local maximum on ChP, *τ*; by tangent to the concentration profile, τ_tan_; by formula (25), *τ*_S_. The results of calculation at *i*_av_/*i*_lim_ = 1.2, 1.25, …, 2.1.

The greater current density, the faster depletion of the concentration in the region near the membrane surface occurs. Therefore, the greater ChP slope angle and less transition time *τ* corresponds to the greater current density *i*_av_ (Figure 7). According to the analytical Sand’s theory, considered the ion transfer in an infinite stagnant diffusion layer at the local electroneutrality assumption, the transition time depends on the inverse square of the current density [29]:

(25) $$\tau_{S}=\frac{\pi \;Pe}{4}\frac{z_{1}^{2}}{{\left(T_{1}-t_{1}\right)}^{2}}\frac{1}{i^{2}}.$$

Calculations at the different values of the current density (*i*_av_/*i*_lim_ = 1.2, 1.25, …, 2.1) show that the time of significant electroconvection development, *τ*, is greater than the depletion time of the concentration to almost zero, *τ*_tan_; and this time (τ_tan_) is greater than the transition time estimated by formula (25), that is, *τ*_S_ < *τ*_tan_ < *τ* (Figure 7).

The discrepancy between *τ*_tan_ and *τ*_S_ is mainly explained by the fact that we consider system with a diffusion boundary layer of a finite thickness, while in Sand’s theory the diffusion layer is infinitely large [45]. 

At *t* = *τ*_tan_ the SCR and, consequently, the bulk force are localized at a relatively small distance from the membrane surface where the viscous forces play the important role due to the non-slip condition. The contribution of EC to intensification of the mass transfer becomes considerable only at *t* = *τ*_tan_. In this case, the SCR thickness increases to become of the order of several micrometers. At such distances, the role of viscous forces decreases.

**Figure 8:**
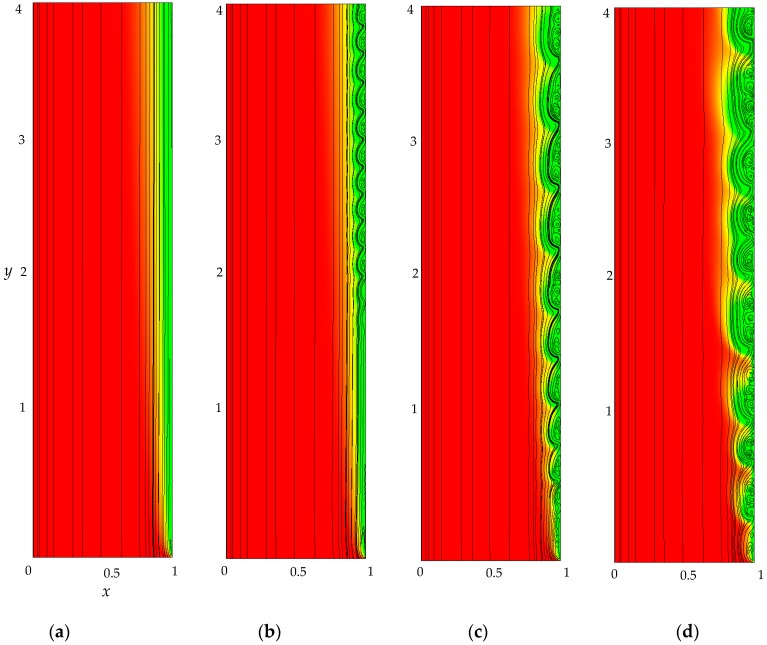
Distribution of cation concentration (the magnitude is shown by different color), solution streamlines (black lines) at *t* = 70, *i*_av_/*i*_lim_=1 (**a**), 1.2 (**b**), 1.5 (**с**), 2 (**d**).

At underlimiting (*i*_av_/*i*_lim_ = 0.9) and limiting (*i*_av_/*i*_lim_ = 1) values of the current density, a monotonic increase in the PD is observed, which slows down with time until it reaches the stationary value $\bar{\Delta \varphi }$. Values of $\bar{\Delta \varphi }$ calculated with and without taking into account ${\vec{f}}_{e}$ differ by less than 0.3% at *i*_av_/*i*_lim_ = 0.9 and 1% at *i*_av_/*i*_lim_ = 1. Electroconvective mixing layer is absent (Figure 8a).

At the overlimiting current density for *t* > *τ*, there is a significant difference in ChP, calculated with and without taking into account ${\vec{f}}_{e}$, which indicates the influence of electroconvective flows on the ion transfer processes (Figure 6a). The behavior of the system is determined by the dynamics and structure of the electroconvective mixing layer (Figure 6b). At current density *i*_av_/*i*_lim_ = 1.2, single vortices (rotating clockwise) are formed along the membrane surface. The forced flow moves the vortices in the tangential direction along the membrane to the channel outlet (Figure 8b). As a result, at *t* > *τ*, the PD oscillates periodically. 

With an increase in the current density, the average thickness of the electroconvective mixing layer, *d*_ec_, also increases (Figure 6b). When *i*_av_/*i*_lim_ = 1.5, the size of the vortices increases, they merge into unstable vortex structures (Figure 8c). On the ChP at *t* > *τ*, oscillations of a greater amplitude and period are observed.

At current density *i*_av_/*i*_lim_ = 2, sizes of the vortex complexes significantly increase, their structure becomes more complicated (Figure 8d). In the quasi-stationary state, the non-periodic large-amplitude oscillations of the PD are observed.

### 3.5. Comparison of the Galvanostatic and Potentiostatic Modes

We compared the results of calculations for the galvanostatic and potentiostatic modes at the same values of the system parameters (given in Section 3.1) and the same computational mesh at current density *i*_av_/*i*_lim_ = 0.9, 1, 1.2, 1.5, 2 (Figure 6). In the potentiostatic mode, instead of condition (18), the PD was set equal to the average value of the PD in the quasi-stationary state, $\bar{\Delta \varphi }$, obtained in the calculation in the galvanostatic mode:

(26) $$\varphi (0,y,t)=0,\;\varphi \left(1,y,t\right)=\bar{\Delta \varphi }.$$

Figure 6c shows time dependences of the average current density, $i_{avp}/i_{\lim}=\frac{1}{l}\int_{0}^{l}\int_{0}^{1}\left(i_{x}/i_{\lim}\right)dxdy$, calculated for the potentiostatic mode (dotted lines). The average current density in the quasi-stationary state (at *t* > 50), calculated for the potentiostatic mode, coincide with the corresponding value of the current density *i*_av_/*i*_lim_ = 0.9, 1, 1.2, 1.5, 2 (values differ by less than 0.6%, see Table 1).

It should be noted that at *t* < *τ*, the PD and current density in the galvanostatic mode are less than in the potentiostatic mode (Figure 6a,c). Therefore, the depletion of the ion concentration, formation of the extended SCR and development of electroconvection occur earlier in the potentiostatic mode than in the galvanostatic (Figure 6b). Furthermore, at the same current density in the quasi-stationary state the average thickness of the electroconvective mixing layer, *d*_ec_, varies in approximately the same range of values for both modes (values differ by less than 7%).

### 3.6. Effect of the System Parameters

In order to estimate the effects of the system parameters on the ion transfer processes in flow-through ED membrane cells, we compare ChP and dynamics of the average thickness of the electroconvective mixing layer, *d*_ec_, calculated at *i*_av_/*i*_lim_ = 2 for various values of the electrolyte solution concentration, *c*_0_, channel length, *l*, channel width, *h*, forced flow velocity, *V*_0_.

The channel length and width, forced flow velocity affect the initial ohmic PD (Equation (23)), therefore the ChP calculated with variation of these parameters are plotted in the coordinates of the corrected PD ∆*φ*’ (∆*φ*’ = ∆*φ* − ∆*φ*_ohm_).

#### 3.6.1. Effect of the Electrolyte Solution Concentration

**Figure 9:**
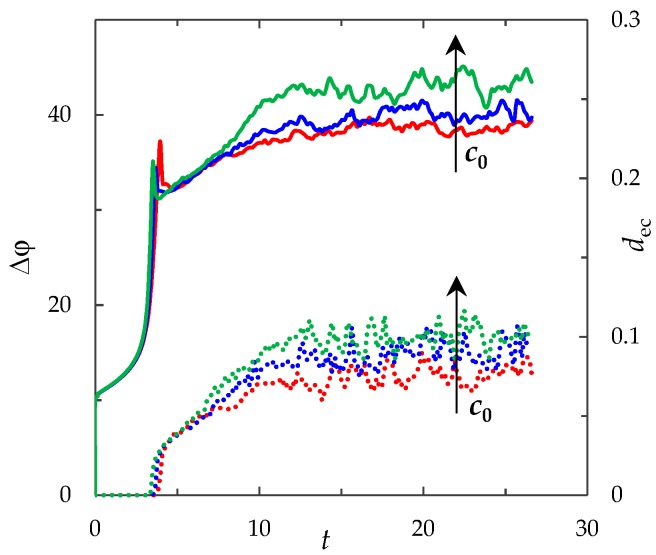
ChP (solid lines) and the average thickness of the electroconvective mixing layer, *d*_ec_ (dotted lines). The results of computation for с_0_ = 0.1 mol/m^3^ (red lines), 0.3 mol/m^3^ (blue lines), 1 mol/m^3^ (green lines) at current density *i*_av_/*i*_lim_ = 2.

The electrolyte solution concentration, *c*_0_, is included in the dimensionless formulation of the model through the *ε*. When *ε* decreases (concentration *c*_0_ increases), the electric field strength increases in the electric double layer. Therefore, when *c*_0_ increases, firstly, the process of electrodiffusion desalination of the electrolyte solution accelerates (the growth rate of the PD increases) and the transition time, *τ*, decreases; secondly, the electric body force increases while the thickness of the extended SCR decreases. To estimate the total effect of these factors on the ion transfer processes, we calculated ChP for the following values of the initial electrolyte concentration: *c*_0_ = 0.1 mol/m^3^ (ε = 3 × 10^−8^), 0.3 mol/m^3^ (ε = 10^−8^), 1 mol/m^3^ (ε = 3 × 10^−9^) at current density *i*_av_/*i*_lim_ = 2 (Figure 9). 

**Figure 10:**
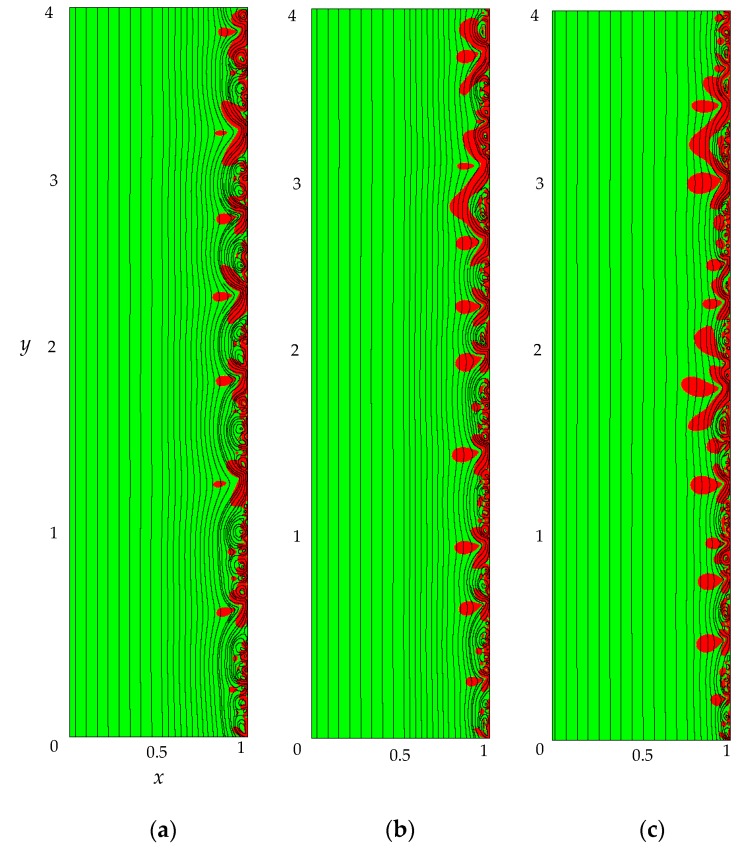
Solution streamlines (black lines) and electroconvective mixing layer (red color) at *t* = 17. The results of calculation for *с*_0_ = 0.1 mol/m^3^ (**a**), 0.3 mol/m^3^ (**b**), 1 mol/m^3^ (**c**) at *i*_av_/*i*_lim_ = 2.

Calculations show that for a greater concentration, a quasi-equilibrium state occurs at a greater value of the average PD, $\bar{\Delta \varphi }$, (Table 2) and a higher intensity of electroconvection (Figure 9). At the same time, the increase in *с*_0_ does not increase the size of the vortices and vortex complexes, but causes the increase in their number (Figure 10).

#### 3.6.2. Effect of the Channel Length

**Figure 11:**
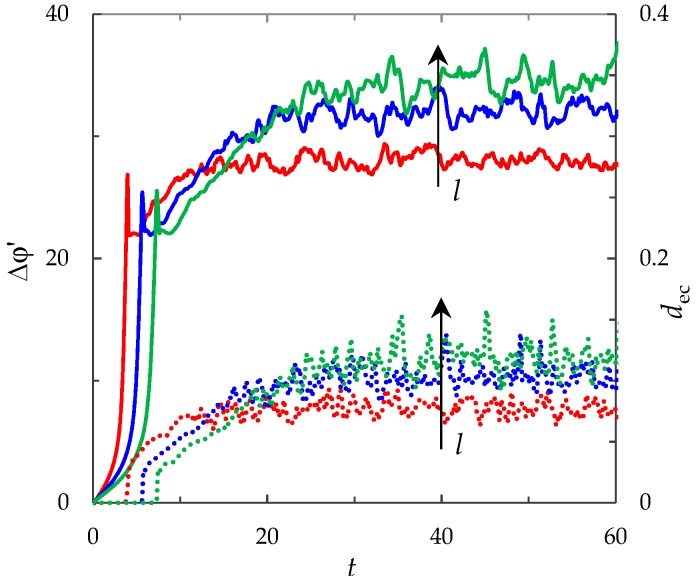
ChP (solid lines) and average thickness of the electroconvective mixing layer, *d*_ec_ (dotted lines). The results of calculation for *l* = 4 (red lines), 8 (blue lines), 12 (green lines) at current density *i*_av_/*i*_lim_ = 2.

Figure 11 shows the ChP and average thickness of the electroconvective mixing layer, *d*_ec_, calculated for the different values of the channel length: *l* = 4, 8, 12 (at the same other parameters). As can be seen from Figure 11, as *l* increases, the transition time, PD and average thickness of the electroconvective mixing layer in the quasi-stationary state increase.

The channel length, *l*, besides defining the considered geometry, also influences a limiting current density, *i*_lim_. With an increase in the channel length, the limiting current density, *i*_lim_, decreases, and therefore the rate of the electrodiffusion desalting process of the electrolyte solution slows down. In this, the electrolyte concentration decreases as the solution moves along the channel with forced flow. Hence, as the channel length increases, the desalting process proceeds more slowly, but the degree of electrolyte desalting reaches higher values. The latter causes the increase in the solution resistance and PD in the quasi-stationary state. The increase in PD causes more intense electroconvection (Figure 11).

#### 3.6.3. Effect of the Channel Width

**Figure 12:**
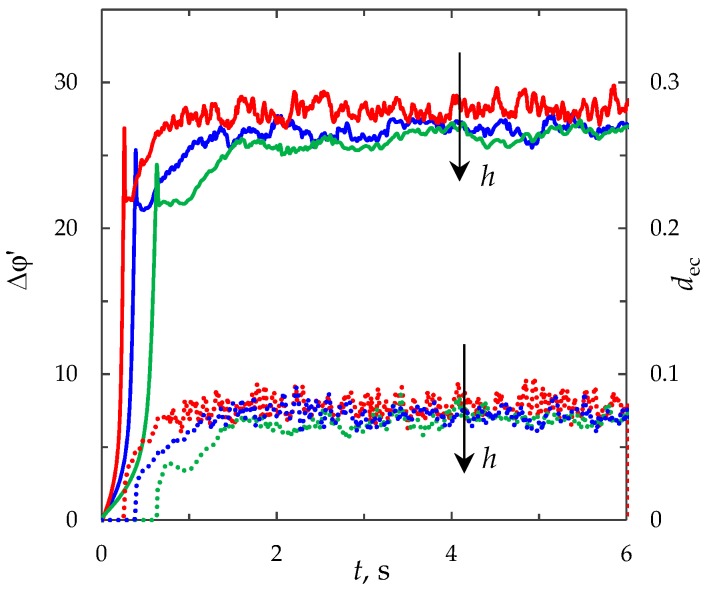
ChP (solid lines) and the average thickness of the electroconvective mixing layer, *d*_ec_ (dotted lines). The results of computation for *h* = 0.25 × 10^−3^ m (red lines), 0.5 × 10^−3^ m (blue lines), 10^−3^ m (green lines) at current density *i*_av_/*i*_lim_ = 2.

The channel width, *h*, is included in all dimensionless numbers of the model. An increase in the channel width causes the increase in the numbers *Pe*, *Re*, *i*_lim_ and the decrease in *ε* and *K*_el_. These facts have different effects on the growth rate of the PD and the intensity of electroconvection. The total effect of the channel width was estimated on the basis of calculations of the ChP and average thickness of the electroconvective mixing layer, *d*_ec_, for different values of the channel width *h* = 0.25 × 10^−3^ m, 0.5 × 10^−3^ m, 10^−3^ m (Figure 12). The dimensional time is used in Figure 12, since the channel width is used during the transition to the dimensionless time. The thickness of the electroconvective mixing layer, *d*_ec_, is normalized by the value *h* = 0.25 × 10^−3^ m in all the calculations. From Figure 12 it can be seen, that the effect of these factors is manifested by the increase in the transient time, decrease in the PD and average thickness of the electroconvective mixing layer in the quasi-stationary state.

#### 3.6.4. Effect of the Forced Flow Velocity

**Figure 13:**
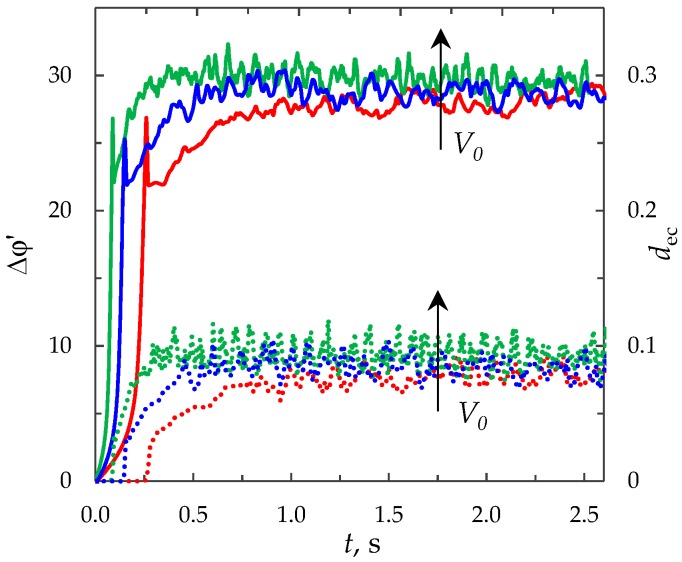
ChP (solid lines) and the average thickness of the electroconvective mixing layer, *d*_ec_ (dotted lines). The results of computation for *V*_0_ = 3.8 × 10^−3^ m/s (red lines), 7.6 × 10^−3^ m/s (blue lines), 15.2 × 10^−3^ m/s (green lines) at current density *i*_av_/*i*_lim_ = 2.

Figure 13 shows the ChP and average thickness of the electroconvective mixing layer, *d*_ec_, calculated for the different values of the forced flow velocity *V*_0_ = 3.8 × 10^−3^ m/s, 7.6 × 10^−3^ m/s, 15.2 × 10^−3^ m/s (at the same other parameters). As in Section 3.6.3, the dimensional time is used in Figure 13. The thickness of the electroconvective mixing layer, *d*_ec_, is determined with the same threshold velocity value, namely, 5% of *V*_0_ = 3.8 × 10^−3^ m/s. 

As can be seen from Figure 13, the total effect of the forced flow velocity is manifested in the decrease in the transient time; the increase in the average PD and thickness of the electroconvective mixing layer in the quasi-stationary state.

### 3.7. Comparison with the Experiment

**Figure 14:**
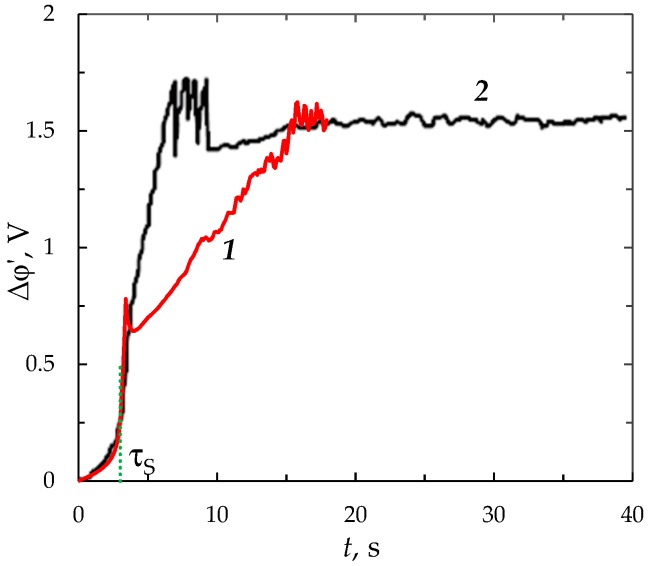
Calculated 1 for *c*_0_ = 0.01 mol/m^3^ (*ε* = 1.53 × 10^−9^) and experimental *2* ChP for a МA-40-13 membrane in a 5 mol/m^3^ NaCl solution (*ε* = 3.06 × 10^−12^) at *i/i*_lim_ = 3.6. The Sand (*τ*_s_ = 3 s) transition times is shown with vertical dotted lines. The experimental data are taken from [44].

Figure 14 shows the ChP obtained experimentally [44] and theoretically using the proposed model for the galvanostatic mode. The experiment was carried out with a anion-exchange membrane МA-40-13 and 5 mol/m^3^ NaCl solution at current density *i*_av_*/i*_lim_ = 3.6; the corresponding value of *ε* is 3 × 10^−12^; the membrane active area was 2 × 2 cm^2^, the intermembrane distance *H* = 7 × 10^−3^ m, the temperature *T* = 293 K. A NaCl solution was flowing between the membranes with an average velocity of *V*_0_ = 3.2 × 10^−3^ m/s; the diffusion coefficients of cations *D*_1_ = 1.33 × 10^−9^ m^2^/s and anions *D*_2_ = 2.05 × 10^−9^ m^2^/s, respectively; the NaCl diffusion coefficient *D* = 1.61 × 10^−9^ m^2^/s; the anion transport number in the membrane *T*_1_ = 1 and that in the solution *t*_1_ = 0.604; the ion charge numbers *z*_1_ = 1, *z*_2_ = −1.

The calculations were performed for the same parameters, only *ε* is 1.5 × 10^−9^ (*c*_0_ = 0.01 mol/m^3^). The increased value of *ε* is taken because of computational complexity, which grows with decreasing *ε*. Calculations for the parameters in Section 3.1 (small channel) at various values of the concentration, *c*_0_, showed that with an increase in *c*_0_ by 10 times (at the same other parameters), the calculation time increases by about 4 times. The calculation ChP for the parameters as in the experiment (large channel) but with *c*_0_ = 0.01 mol/m^3^ from 0 to 15 s took approximately 150 h. Thus, calculations for the parameters as in the experiment (large channel and concentration of 5 mol/m^3^) will take several months. 

In Figure 14, the reduced PD is used, since it allows to exclude the influence of ohmic resistance, which is a function of the distance between the measuring electrodes, the membrane thickness, and some other parameters that are difficult to find [44], whereas they are not significant for the membrane behavior and are not taken into account in the model.

Theoretical and experimental curves are characterized by similar behavior: monotonous slow growth of the PD, transitional stage of electroconvection development, quasi-stationary state. In these conditions, the transition time *τ* computed using the model is equal to 3.4 s. The experimental transition time $\tau_{\exp}$ found by inflection point is 3.8 s. A good agreement between experimental and calculated curves is observed at times *t* < *τ*_S_ = 3 s (the difference of experimental and calculated values of the PD is less than 10%). The transitional stage of electroconvection development differs more significantly. On the calculated CP at time *τ*, a sharp decrease in the PD is observed. In the experimental CP, the growth rate of the PD decreases smoothly due to the appearance of additional mechanisms, the main of which is electroconvection [44]. Apparently this is due to the geometrical and electrical heterogeneity of the membrane surface [46,47], which influences the development of electroconvection. While, in the experiment, the membrane surface is assumed to be perfectly homogeneous. For a more accurate description of ChP of systems with ion-exchange membranes, the proposed 2D modelling should be extended by the introduction into the model tools describing the heterogeneity of the membrane surface and by transition to the 3D case.

## 4. Discussion

Mathematical modelling of 2D non-stationary ion transfer in flow-through ED membrane cells in the galvanodynamic mode is carried out using the Navier–Stokes, Nernst–Planck and Poisson equations. Half of the desalination channel at the cation-exchange membrane is considered. For setting the electric field mode, the boundary condition on the electric field strength and method of the electric current stream function are used. The developed model allows us to describe the formation of the extended SCR and development of electroconvection at overlimiting currents. For the first time, theoretical ChP and CVC of membrane systems taking into account the forced flow and electroconvection are obtained. The behaviors of the calculated ChP and CVC coincide qualitatively with the experimental data.

A comparison of the results for the galvanostatic and potentiostatic modes showed that the depletion of the ion concentration, formation of the extended SCR and development of electroconvection occur earlier in the potentiostatic mode than in galvanostatic. The results of calculation of PD/current density in quasi-steady state using galvanostatic/potentiostatic models are in good agreement.

It is found that an increase in the electrolyte concentration, channel length and velocity of the forced flow increases the intensity of electroconvection and the PD in the quasi-stationary state. With an increase in the channel width, there is a decrease in the PD and average thickness of the electroconvective mixing layer in the quasi-stationary state.

Understanding the mechanisms of transfer processes at a fixed current density is important for obtaining a fundamental insight and for optimizing the operation practice of flow-through membrane systems.

## Figures and Tables

**Table 1:** The potential drop (PD) and average current density in the quasi-stationary state in the galvanostatic and potentiostatic modes.

$i_{\mathrm{av}}/i_{\lim}$	$\bar{\Delta \varphi }$	$\bar{i_{avp}}/i_{\lim}$
0.9	8.5	0.900
1	12.2	1.000
1.2	25.6	1.201
1.5	30.6	1.499
2	38.9	2.012

**Table 2:** The transition time and PD in the quasi-stationary state.

*с*_0_, mol/m^3^	ε	*τ*	$\bar{\Delta \varphi }$
0.1	3 × 10^−8^	3.95	38.8
0.3	10^−8^	3.65	40.2
1	3 × 10^−9^	3.50	43.0
